# Assessment of Plasma Amyloid-β_42/40_ and Cognitive Decline Among Community-Dwelling Older Adults

**DOI:** 10.1001/jamanetworkopen.2020.28634

**Published:** 2020-12-17

**Authors:** Kelly Virecoulon Giudici, Philipe de Souto Barreto, Sophie Guyonnet, Yan Li, Randall John Bateman, Bruno Vellas

**Affiliations:** 1 Gerontopole of Toulouse, Institute of Ageing, Toulouse University Hospital, Toulouse, France; 2 UPS/Inserm UMR1027, University of Toulouse III, Toulouse, France; 3 Department of Neurology, Washington University School of Medicine in St Louis, St Louis, Missouri; 4 Division of Biostatistics, Washington University School of Medicine in St Louis, St Louis, Missouri

## Abstract

**Importance:**

Plasma measurement of amyloid-β (Aβ) peptides has been associated with cognitive function, but evidence of its ability to identify cognitive decline is still scarce.

**Objective:**

To investigate the associations between plasma Aβ_42/40_ and cognitive decline over time among community-dwelling older adults with subjective memory concerns.

**Design, Setting, and Participants:**

This multicenter cohort study used data from volunteers in the 5-year study Multidomain Alzheimer Preventive Trial (MAPT). Participants were aged 70 years or older and observed for a median (interquartile range) of 3.9 (2.0-4.0) years. Recruitment of participants started in May 2008 and ended in February 2011. Follow-up ended in April 2016. Data analysis was conducted from April to October 2020.

**Exposure:**

Plasma Aβ_42_ and Aβ_40_ were measured at 12 months for 448 participants (92.8%) and at 24 months for the rest. The moment of Aβ assessment was defined as the baseline for this study.

**Main Outcomes and Measures:**

Cognitive function was assessed at 12, 24, 36, 48, and 60 months by a composite cognitive score based on 4 tests; Mini Mental State Examination (MMSE); Clinical Dementia Rating, sum of boxes; and Alzheimer Disease Cooperative Study–Activities of Daily Living. Mixed-effect linear regressions were performed.

**Results:**

A total of 483 participants (median [IQR] age, 76.0 [73.0-80.0]; 286 [59.2%] women) were analyzed. Of them, 161 (33.3%) were classified as low plasma Aβ_42/40_ (≤0.107). After adjusting for age, sex, education, body mass index, Geriatric Depression Scale score, apolipoprotein E ε4 genotype, and MAPT intervention groups, low plasma Aβ_42/40_ was associated with more pronounced decline in composite cognitive score (adjusted between-group mean difference: −0.20, 95% CI, −0.34 to −0.07; *P* = .004) and decline in MMSE score (adjusted between-group mean difference: −0.59; 95% CI, −1.07 to −0.11; *P* = .02) during the follow-up period compared with the group with an Aβ_42/40_ ratio greater than 0.107.

**Conclusions and Relevance:**

In this study, low plasma Aβ_42/40_ was associated with more pronounced decline in cognitive function (measured by multiple outcomes) over time. Findings suggest that plasma Aβ_42/40_ may be used to identify people at risk of cognitive decline, being an alternative to more complex and expensive measures, such as positron emission tomography imaging or cerebrospinal fluid measurement.

## Key Points

**Question:**

Is plasma amyloid-β_42/40_ (Aβ_42/40_) associated with cognitive decline among community-dwelling older adults with memory concerns?

**Findings:**

In this cohort study of 483 participants from a randomized clinical trial, low plasma Aβ_42/40_ ratio was significantly associated with more pronounced decline in composite cognitive score and Mini Mental State Examination score over time.

**Meaning:**

In this study, low plasma Aβ_42/40_ was associated with more pronounced decline in cognitive function over time, suggesting that this marker may be used to identify people at risk of cognitive decline and as an alternative to more complex and expensive measures.

## Teaser

This cohort study investigates the associations between plasma amyloid-β_42/40_ and cognitive decline over time among community-dwelling older adults with subjective memory concerns.

## Introduction

Brain accumulation of amyloid-β (Aβ) peptides is known to be intimately associated with the physiological landscape of Alzheimer disease (AD).^1^ Measures of Aβ have been used as an early marker of cognitive impairment and AD, assessed by positron emission tomography (PET) imaging or measurement in cerebrospinal fluid (CSF).^1^ In a search for less expensive, minimally invasive, and fast and reliable markers, plasma measures of Aβ have recently emerged as a potential equivalent to PET imaging and CSF measurements in determining Aβ status.^2,3,4,5^ Early attempts to measure Aβ in plasma presented limited utility for diagnosis or prognosis of cognitive impairment and AD due to high variability attributed to a lack of high precision methods of assessment in plasma samples.^5,6,7^ More recently, a high-precision immunoprecipitation and liquid chromatography–mass spectrometry assay has provided reliable measures of plasma Aβ peptides,^2,3,8,9,10,11^ but investigations associating this marker with clinical cognitive outcomes are scarce.

The association between plasma Aβ and cognitive function has been previously shown in cross-sectional^12^ and longitudinal analyses^13,14,15,16^; however, such publications from approximately a decade ago provided low accuracy for plasma Aβ measures at the individual level. Studies exploring cognitive associations with longitudinal cohorts of older adults by using highly reliable techniques are still scarce and present multiple methodological differences that prevented reaching a consensus.^17,18,19,20^ Further studies are needed to confirm the use of high-accuracy plasma Aβ in associating Aβ levels with cognitive decline to determine the usefulness of this marker in clinical care and research.

This study aimed to investigate the associations between plasma Aβ_42/40_ and cognitive decline over time among community-dwelling older adults with spontaneous memory concerns. We hypothesized that Aβ_42/40_ status may be associated with changes in cognitive function over time among community-dwelling older adults, with lower Aβ ratio associated with more pronounced cognitive decline.

## Methods

### Study Design and Population

This cohort study uses data from participants from the Multidomain Alzheimer Preventive Trial (MAPT), a randomized, multicenter, placebo-controlled trial conducted with community-dwelling older adults in France and Monaco. Participants were allocated into 4 groups, either receiving ω-3 polyunsaturated fatty acid (PUFA) supplementation, a multidomain intervention (based on cognitive training, nutritional counseling, and physical activity advice), both, or none (in this case, taking placebo capsules). The intervention phase lasted for 3 years and was then followed by an additional 2-year observational phase (without any intervention). Recruitment of participants started in May 2008 and ended in February 2011. Follow-up ended in April 2016.

**Figure 1.**
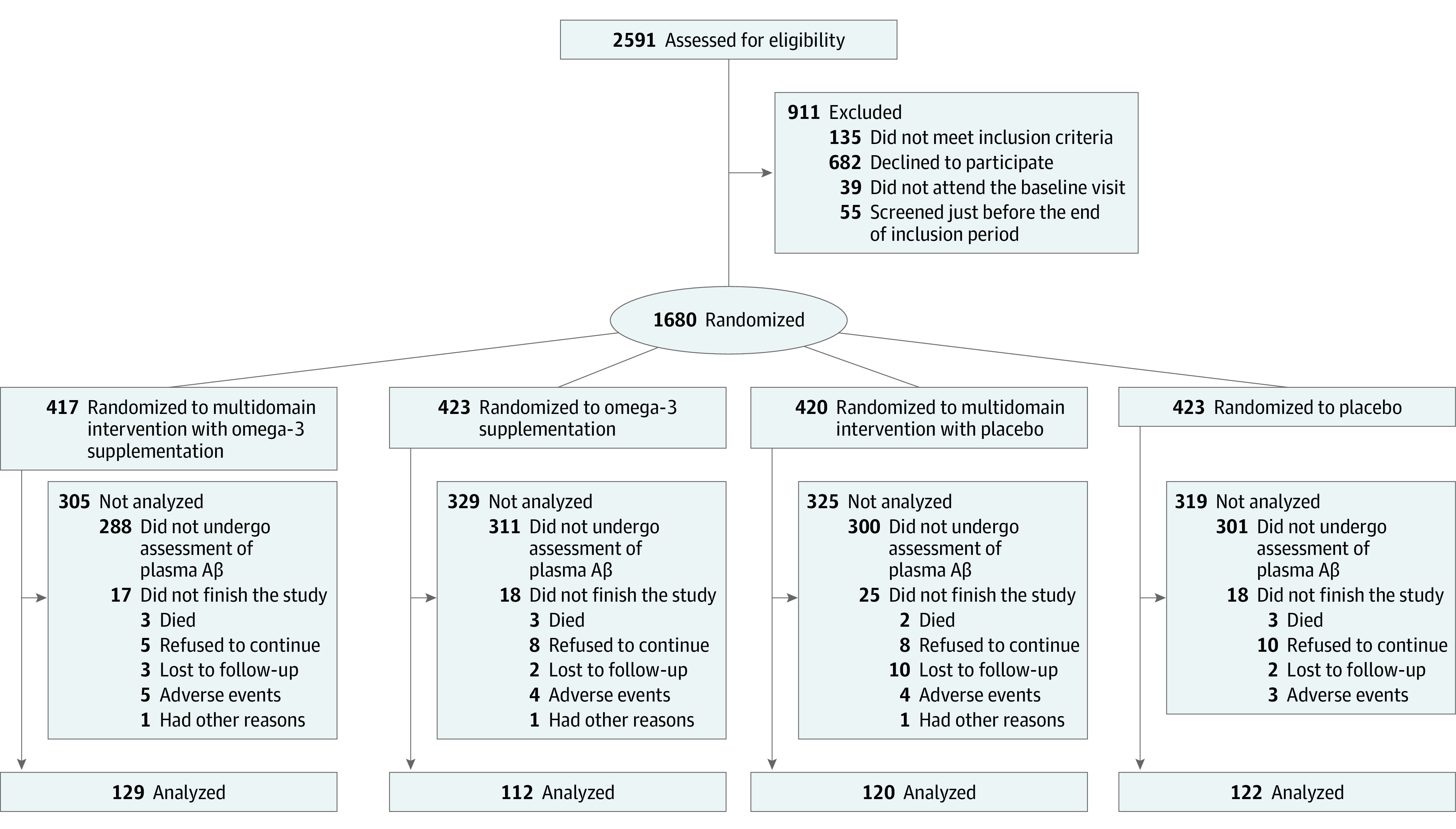
Flow Diagram Describing the Population of the Study Aβ indicates amyloid-β.

Complete inclusion and exclusion criteria as well as other details about the MAPT protocol, can be found elsewhere.^21,22^ In summary, inclusion criteria comprised age 70 years or older; not presenting major neurocognitive disorders (Mini-Mental State Examination [MMSE] score, ≥24); presenting at least 1 of the following: spontaneous memory concern, inability to perform 1 instrumental activity of daily living (ADL), or slow walking speed (<0.8 m/s in a 4-m usual walking test). Participants were not included if they declared the use of ω-3 PUFA supplements during the 6 months prior to inclusion. From the 1680 individuals originally included in MAPT, 483 with available blood samples had their plasma Aβ measured and were thus included in the present study (Figure 1). A comparison of baseline characteristics between MAPT participants enrolled in the present study and those who were not included is shown in eTable 1 in the Supplement.

### Ethical Disclosure

Eligible subjects provided written informed consent after accepting to join the investigation. The MAPT Study (trial protocol NCT00672685) was authorized by the French Health Authority and approved by the Advisory Committee for the Protection of Persons participating in Biomedical Research of Toulouse. The present study followed the Strengthening the Reporting of Observational Studies in Epidemiology (STROBE) reporting guideline.^23^

### Aβ Status

Plasma Aβ_42_ and Aβ_40_ were measured at 12 months for 448 participants (92.8%) and at 24 months for 35 (7.2%) (due to unavailability of samples at baseline and 12 months). Plasma samples were spiked with a known quantity of 12C15N-Aβ_42_ and 12C15N-Aβ_40_ for use as analytical internal standards. A full description of the immunoprecipitation methods applied has been previously described.^11^ Briefly, Aβ_42_ and Aβ_40_ isoforms were simultaneously immunoprecipitated from 0.45 mL of plasma via a monoclonal anti-Aβ middomain antibody (HJ5.1, anti-Aβ13-28) conjugated to M-270 Epoxy Dynabeads (Invitrogen). Protein digestion into peptides was done using LysN endoprotease (Pierce). Liquid chromatography–mass spectrometry was performed as detailed elsewhere.^11^ Plasma analyses were performed as targeted parallel reaction monitoring on an Orbitrap Fusion Lumos Tribrid mass spectrometer (Thermo Fisher) interfaced with an M-class nanoAcquity chromatography system (Waters). The precursor and product ion pairs used for analysis of Aβ isoforms were chosen as previously detailed.^2,24^ Derived integrated peak areas were analyzed using the Skyline software package.^25^ Aβ_42_ and Aβ_40_ quantities (in picograms per milliliter) were calculated by integrated peak area ratios to known concentrations of the internal standards. The plasma Aβ_42/40_ ratio was then calculated by dividing Aβ_42_ by Aβ_40_, and its normalized values were used to classify Aβ status (determined by Youden index as low if ≤0.107 and normal if >0.107, using amyloid PET status as the reference standard).

### Outcomes

Outcomes were measured annually and comprised a composite cognitive score based on 4 tests; the MMSE score; the Clinical Dementia Rating (CDR) sum of boxes; and the Alzheimer Disease Cooperative Study–ADL (ADCS-ADL) score. The composite cognitive score (whose higher values mean better cognitive function) was composed of the mean value of 4 *z *scores, given by the 10 orientation items of the MMSE, the Digit Symbol Substitution Test, free and total recall of the Free and Cued Selective Reminding test, and the Category Naming Test.^22^ The MMSE score ranges from 0 to 30, with higher scores indicating better function.^26^ The CDR sum of boxes evaluates 6 domains (memory; orientation; judgement and problem solving; community affairs; home and hobbies; and personal care), which are scored individually from 0 to 3 (thus achieving a maximum score of 18, with higher scores indicating worse function).^27^ Finally, the ADCS-ADL scale ranges from 0 to 45, with higher scores indicating better function.^28^

### Potential Confounders

Potential confounders consisted of age (continuous variable), sex (male vs female), education (no diploma, primary school certificate, secondary education, high school diploma, university level), body mass index (BMI; calculated as weight in kilograms divided by height in meters squared), allocation to MAPT groups (multidomain intervention with ω-3 supplementation; multidomain intervention with placebo; ω-3 supplementation alone; and placebo alone), CDR status at baseline (CDR score 0, 0.5, or ≥1), Geriatric Depression Scale (GDS) score (continuous), and apolipoprotein E (APOE) ε4 genotype (carrier vs noncarrier).

### Statistical Analysis

Descriptive statistics (medians and interquartile ranges [IQRs] or frequencies and percentages, as appropriate) were used for characterization of the study population. The moment in which participants had their plasma Aβ measured was considered the baseline (ie, either 12 or 24 months, as appropriate); no outcome data obtained before Aβ measurement were used. Quantitative variables at baseline were compared according to Aβ status by Wilcoxon-Mann-Whitney test, and categorical variables were compared using the χ^2^ test.

Linear mixed-effects regression analyses were performed to determine associations between plasma Aβ status (independent variable) and changes in outcomes (dependent variables) over time, with adjustments for potential confounders (model 1: sex, age, education, BMI, MAPT group, CDR status at baseline, GDS score, and APOE ε4 genotype; model 2: all confounders except APOE ε4 genotype). A Cramer V of −0.20 indicated weak collinearity between APOE ε4 genotype and Aβ status. CDR status at baseline was not included when the outcome was CDR sum of boxes. In the absence of an agreed-upon range in literature to determine plasma Aβ status, sensitivity analyses using the 25th percentile of Aβ_42/40_ as an alternative cutoff were performed (low Aβ, ≤0.103). In addition, to rule out the potential effects of MAPT interventions on both Aβ and cognitive outcomes, sensitivity analyses using the cutoff of 0.107 but restricted to the placebo group (n = 122) were done. Sensitivity analyses were also performed with plasma Aβ as a continuous variable, including the same potential confounders as the models reported earlier. For these analyses, 1 participant was excluded due to presenting extremely high value of Aβ_42/40_ (>12 SDs above the mean value).

Cox proportional hazard models were performed to explore associations between plasma Aβ_42/40_ and worsening CDR status among participants with CDR scores of less than 1 at baseline, considering the same models of adjustment already described. Time to first event was determined as the time interval for changing from cognitively normal (CDR score, 0) at baseline to mild cognitive impairment (MCI; CDR score, 0.5) or changing from MCI at baseline to major cognitive impairment (CDR score, ≥1). Participants without the event were censored at their last CDR assessment visit. Proportional hazard assumption was tested using the Kolmogorov-type supremum test, and *P* > .05 was considered nonviolation of the assumption. Analyses were performed with the SAS version 9.4 (SAS Institute), at a significance level of *P* < .05 with 2-tailed tests. Data analysis was conducted from April to October 2020.

## Results

### Characterization of the Sample

From the 483 participants of the study (median [IQR] age, 76.0 [73.0-80.0] years), 286 (59.2%) were women and 128 (26.9%) had a university-level education. As presented in Table 1, 161 participants (33.3%) were classified as having low plasma Aβ_42/40_ (≤0.107; hereafter, Aβ+). The Aβ+ group, compared with participants in the Aβ– group (ie, those with Aβ_42/40_ >0.107), was older (median [IQR] age, 77.0 [73.0-80.0] years vs 76.0 [73.0-80.0] years; *P* = .02) and included more men (80 [49.7%] vs 117 [36.3%]; *P* = .005) and more APOE ε4 carriers (60 [40.3%] vs 61 [21.1%]; *P* < .001). Median (IQR) follow-up was 3.9 (2.0-4.0) years.

**Table 1.** Characteristics of the Sample According to Plasma Aβ_42/40_ Status

Characteristics	Participants, No.	Median (IQR)		
		Total **(**N = 483**)**	Low plasma Aβ_42/40_ **(**n = 161**)**^a^	Normal plasma Aβ_42/40_ **(**n = 322**)**
Women, No. (%)	483	286 (59.2)	81 (50.3)	205 (63.7)^b^
Age, y	483	76.0 (73.0-80.0)	77.0 (73.0-80.0)	76.0 (73.0-80.0)^b^
Education, No. (%)				
No diploma	476	22 (4.6)	6 (3.8)	16 (5.1)
Primary school certificate		99 (20.8)	39 (24.5)	60 (18.9)
Secondary education		158 (33.2)	61 (38.4)	97 (30.6)
High school diploma		69 (14.5)	16 (10.1)	53 (16.7)
University level		128 (26.9)	37 (23.3)	91 (28.7)
Weight, kg	480	69.3 (61.0-79.0)	70.0 (61.0-79.0)	69.0 (61.0-79.0)
Body mass index^c^	480	26.0 (23.6-28.7)	25.8 (23.9-28.2)	26.2 (23.5-28.8)
Plasma amyloid-β_42/40_ ratio	483	0.113 (0.103-0.123)	0.099 (0.093-0.103)	0.119 (0.113-0.127)^b^
Composite cognitive score^d^	478	0.16 (−0.28 to 0.55)	0.10 (−0.45 to 0.53)	0.17 (−0.25 to 0.56)
CDR sum of boxes, range 0-18	481	0.5 (0 to 0.5)	0.5 (0 to 0.5)	0.5 (0 to 0.5)
CDR status, No. (%)				
No cognitive impairment, CDR score, 0	481	212 (43.9)	64 (39.8)	148 (46.0)
Mild cognitive impairment, CDR score, 0.5		268 (55.5)	96 (59.6)	172 (53.4)
Major cognitive impairment, CDR score, ≥1		3 (0.6)	1 (0.6)	2 (0.6)
MMSE score, range 0-30	481	28 (27-29)	28 (26-29)	28 (27-29)
ADCS-ADL score, range 0-45	473	41 (37-44)	41 (37-43)	41 (37-44)
Geriatric Depression scale, range 0-15	479	2 (1-4)	3 (1-4)	2 (1-5)
APOE ε4 genotype, No. (%)				
APOE ε4 carriers	438	121 (27.6)	60 (40.3)	61 (21.1)^b^
Non-APOE ε4 carriers		317 (72.4)	89 (59.7)	228 (78.9)

Abbreviations: Aβ, amyloid-β; ADCS-ADL, Alzheimer Disease Cooperative Study–Activities of Daily Living; APOE, apolipoprotein E; CDR, Clinical Dementia Rating; IQR, interquartile range; MMSE, Mini-Mental State Examination.

^a^ Low Aβ_42/40_ defined as 0.107 or less.

^b^ *P* < .05 based on Wilcoxon-Mann-Whitney test or Pearson χ^2^ test.

^c^ Body mass index calculated as weight in kilograms divided by height in meters squared.

^d^ Based on the *z* score of 4 cognitive tests (free and total recall of the Free and Cued Selective Reminding test, 10 MMSE orientation items, Digit Symbol Substitution Test, and Category Naming Test).

### Changes in Outcomes Over Time According to Plasma Aβ Status

**Figure 2.**
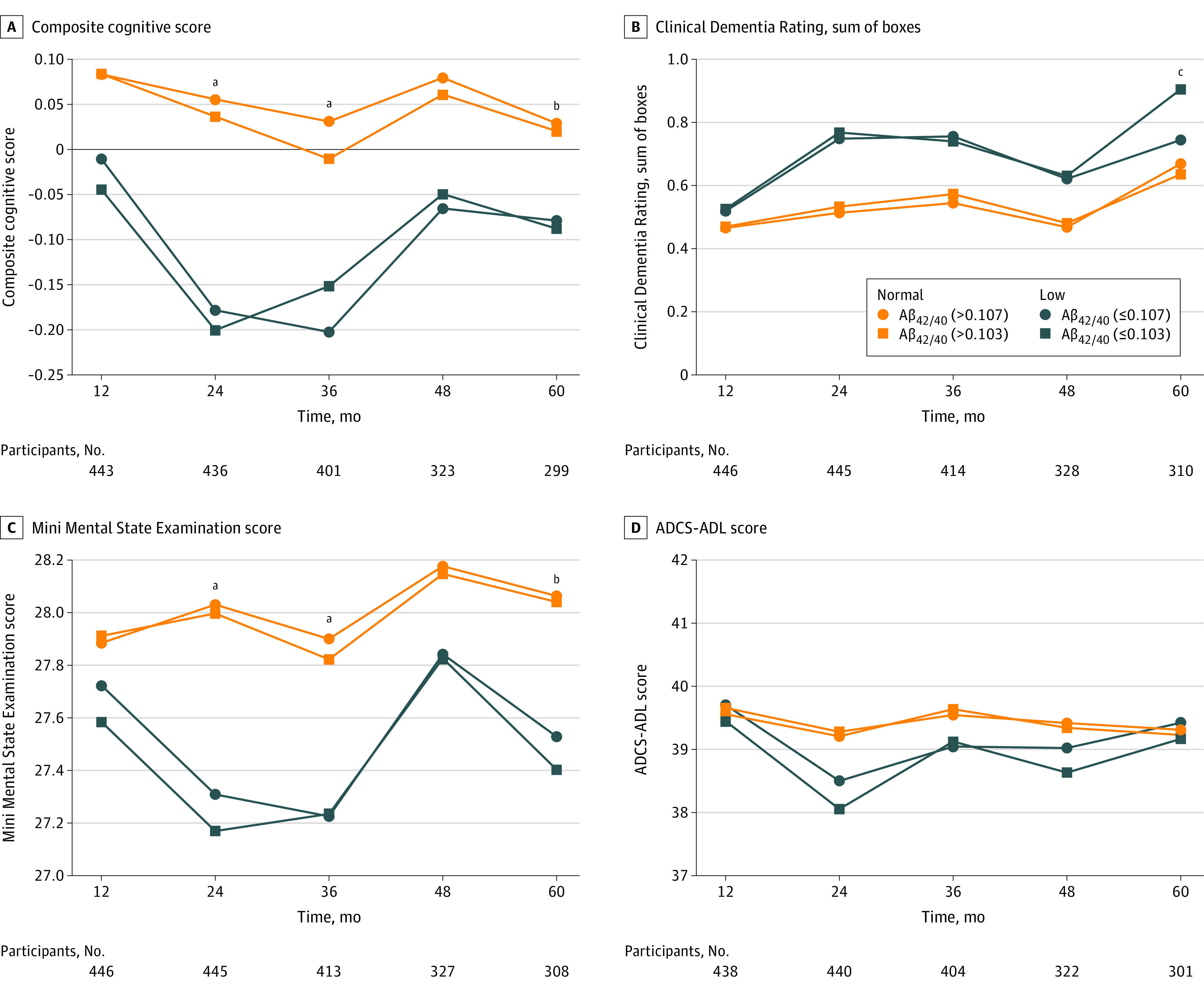
Variation in Outcomes Over Time According to Plasma Amyloid-β_42/40_ Status Among Community-Dwelling Older Adults ADCS-ADL indicates Alzheimer Disease Cooperative Study–Activities of Daily Living. ^a^*P* < .05 for adjusted between-group difference according to the cutoff of 0.107. ^b^*P* < .05 for adjusted between-group difference according to the cutoff of 0.103. ^c^*P* < .05 for adjusted between-group difference according to the cutoffs of 0.107 and 0.103.

During follow-up, both groups experienced significant decrease in composite cognitive score and increase in CDR sum of boxes. Cognitive decline according to the composite cognitive score was more pronounced in the Aβ+ group than in the Aβ– group (adjusted between-group mean difference: −0.20, 95% CI, −0.34 to −0.07; *P* = .004) (Table 2). In the same period, MMSE score significantly decreased in the Aβ+ group and remained stable among Aβ– participants, with a significant difference between groups (adjusted between-group mean difference: −0.59; 95% CI, −1.07 to −0.11; *P* = .02). Both groups presented significant decreases in ADCS-ADL score over time, but there was no significant between-group difference (Table 2 and Figure 2). Adjusted models not including APOE ε4 as a potential confounder provided similar findings (eTable 2 in the Supplement).

**Table 2.** Mixed-Effect Linear Regression Analysis for Variation in Outcomes Over Time According to Plasma Amyloid-β_42/40_ Status Among Community-Dwelling Older Adults

Period	Low plasma amyloid-β_42/40_^a^		Normal plasma amyloid-β_42/40_		Unadjusted model^b^		Adjusted model^c^	
	Estimated mean (95% CI)^d^	*P* value	Estimated mean (95% CI)^d^	*P* value	Differences (95% CI)	*P* value	Differences (95% CI)	*P* value
**Composite cognitive score**^**e**^								
24 mo (1-y change)	−0.25 (−0.33 to −0.16)	<.001	−0.12 (−0.19 to −0.06)	<.001	−0.12 (−0.23 to −0.02)	.03	−0.12 (−0.23 to 0.00)	.04
36 mo (2-y change)	−0.35 (−0.44 to −0.26)	<.001	−0.16 (−0.22 to −0.09)	<.001	−0.19 (−0.30 to −0.08)	.001	−0.19 (−0.30 to −0.07)	.002
48 mo (3-y change)	−0.38 (−0.48 to −0.28)	<.001	−0.19 (−0.26 to −0.12)	<.001	−0.19 (−0.31 to −0.07)	.002	−0.18 (−0.32 to −0.05)	.01
60 mo (4-y change)	−0.45 (−0.56 to −0.35)	<.001	−0.26 (−0.33 to −0.19)	<.001	−0.20 (−0.32 to −0.07)	.002	−0.20 (−0.34 to −0.07)	.004
**CDR sum of boxes, range 0-18**								
24 mo (1-y change)	0.26 (0.10 to 0.41)	.002	0.08 (−0.04 to 0.19)	.18	0.18 (−0.02 to 0.37)	.07	0.11 (−0.08 to 0.30)	.24
36 mo (2-y change)	0.31 (0.15 to 0.48)	<.001	0.12 (0.01 to 0.24)	.04	0.19 (−0.01 to 0.39)	.06	0.18 (−0.02 to 0.38)	.08
48 mo (3-y change)	0.29 (0.10 to 0.47)	.002	0.10 (−0.02 to 0.22)	.11	0.19 (−0.04 to 0.41)	.10	0.12 (−0.11 to 0.34)	.30
60 mo (4-y change)	0.43 (0.24 to 0.62)	<.001	0.29 (0.16 to 0.41)	<.001	0.15 (−0.08 to 0.37)	.21	0.22 (0.01 to 0.44)	.06
**MMSE score, range 0–30**								
24 mo (1-y change)	−0.47 (−0.78 to −0.16)	.003	0.03 (−0.20 to 0.25)	.82	−0.50 (−0.88 to −0.11)	.01	−0.42 (−0.82 to −0.02)	.04
36 mo (2-y change)	−0.69 (−1.01 to −0.36)	<.001	−0.11 (−0.34 to 0.12)	.34	−0.57 (−0.97 to −0.18)	.004	−0.54 (−0.96 to −0.12)	.01
48 mo (3-y change)	−0.37 (−0.74 to −0.01)	.005	−0.01 (−0.25 to 0.23)	.94	−0.36 (−0.80 to 0.07)	.10	−0.30 (−0.78 to 0.18)	.22
60 mo (4-y change)	−0.72 (−1.10 to −0.35)	<.001	−0.16 (−0.41 to 0.08)	.20	−0.56 (−1.01 to −0.11)	.01	−0.59 (−1.07 to −0.11)	.02
**ADCS-ADL score, range 0-45**								
24 mo (1-y change)	−1.39 (−2.16 to −0.62)	<.001	−0.50 (−1.05 to 0.06)	.08	−0.89 (−1.84 to 0.06)	.06	−0.54 (−1.53 to 0.45)	.29
36 mo (2-y change)	−1.28 (−2.09 to −0.48)	.002	−0.22 (−0.78 to 0.35)	.46	−1.07 (−2.05 to −0.08)	.03	−0.95 (−1.98 to 0.09)	.07
48 mo (3-y change)	−1.99 (−2.90 to −1.07)	<.001	−0.61 (−1.21 to −0.01)	.05	−1.37 (−2.47 to −0.28)	.01	−0.92 (−2.11 to 0.27)	.13
60 mo (4-y change)	−1.73 (−2.67 to −0.78)	<.001	−0.85 (−1.46 to −0.24)	.006	−0.88 (−2.00 to 0.25)	.13	−0.34 (−1.54 to 0.86)	.58

Abbreviations: ADCS-ADL, Alzheimer Disease Cooperative Study–Activities of Daily Living; CDR, Clinical Dementia Rating; MMSE, Mini-Mental State Examination.

^a^ Low Aβ_42/40_ defined as 0.107 or less.

^b^ Included 481 participants.

^c^ Included 433 participants. Model was adjusted by age, sex, education, body mass index, apolipoprotein E ε4 genotype, Geriatric Depression Scale score, MAPT intervention group, and CDR status at baseline (except for the analysis with CDR sum of boxes).

^d^ Outcome evolution was compared considering the moment when plasma amyloid-β was measured as baseline (12 months for 448 participants [92.8%] and 24 months for 35 [7.2%]). Negative values for within-group differences mean cognitive decline, except for CDR sum of boxes (for which it is given by positive values). Positive values for between-group differences indicate more pronounced cognitive decline among the low plasma Aβ_42/40_ group, except for CDR sum of boxes (for which it is given by negative values).

^e^ Based on the *z *score of 4 cognitive tests (free and total recall of the Free and Cued Selective Reminding test; 10 MMSE orientation items; Digit Symbol Substitution Test; and Category Naming Test).

From the 212 participants who had CDR scores of 0 at blood assessment and had at least 1 other longitudinal measure of CDR score, 141 (66.5%) evolved to MCI. From the 268 participants who had MCI (CDR score, 0.5) at blood assessment and had at least 1 other longitudinal measure of CDR score, 19 (7.1%) evolved to major cognitive impairment during follow-up. Frequency of events was, therefore, 160 of 459 (34.9%). Participants in the Aβ+ group (according to the main cutoff of 0.107) did not present a significantly higher hazard of CDR worsening, compared with those in the Aβ– group (Table 3).

**Table 3.** Cox Proportional Hazard Models for Clinical Dementia Rating Worsening According to Plasma Aβ_42/40_ Status During Follow-up Period^a^

Group	Unadjusted model (n = 459)		Adjusted			
			Model 1 (n = 410)^b^		Model 2 (n = 450)^c^	
	HR (95% CI)	*P* value	HR (95% CI)	*P* value	HR (95% CI)	*P* value
Predefined cutoff						
Normal plasma Aβ_42/40_ (>0.107)	1 [Reference]	NA	1 [Reference]	NA	1 [Reference]	NA
Low Aβ_42/40_ (≤0.107)	1.09 (0.79-1.52)	.60	1.03 (0.71-1.49)	.89	1.02 (0.72-1.44)	.91
**Alternative cutoff, ie, lowest quartile**						
Normal plasma Aβ_42/40_ (>0.103)	1 [Reference]	NA	1 [Reference]	NA	1 [Reference]	NA
Low Aβ_42/40_ (≤0.103)	1.36 (0.97-1.92)	.08	0.97 (0.67-1.42)	.89	0.98 (0.69-1.39)	.91

Abbreviation: HR, hazard ratio; NA, not applicable.

^a^ CDR worsening was defined as changing from cognitively normal (CDR score, 0) at baseline to mild cognitive impairment (CDR score, 0.5) or changing from mild cognitive impairment at baseline to major cognitive impairment (CDR score, ≥1) considering the moment when plasma Aβ was measured as the baseline (12 or 24 months).

^b^ Model 1 was adjusted by age, sex, education, body mass index, Geriatric Depression Scale score, apolipoprotein E ε4 genotype, and MAPT intervention group.

^c^ Model 2 was adjusted by age, sex, education, body mass index, Geriatric Depression Scale score, and MAPT intervention group.

### Sensitivity Analyses With Lowest Aβ_42/40_ Quartile as Cutoff

Using the lowest quartile to classify Aβ status resulted in 120 participants (24.8%) categorized as Aβ+ (Aβ_42/40_, ≤0.103). This group, compared with participants with Aβ_42/40_ greater than 0.103, was older (median [IQR] 77.0 [73.0-80.5] years vs 76.0 [73.0-80.0] years; *P* = .02) and included fewer women (57 [47.5%] vs 229 [63.1%]; *P* = .003) and more APOE ε4 carriers (48 [41.7%] vs 73 [22.6%]; *P* < .001). Analyses of the evolution of outcomes over time according to this alternative classification are shown in eTable 3 in the Supplement. Findings were similar to those presented with the original cutoff: participants in the Aβ+ group presented a more pronounced decline in composite cognitive score; MMSE only declined among the Aβ+ group. In addition, a more pronounced increase in CDR sum of boxes was observed in this group. Cox analysis using the alternative cutoff of 0.103 for Aβ status found no difference in hazard of CDR worsening among participants in Aβ+ and Aβ– groups (Table 3).

### Sensitivity Analysis: Restricted to the MAPT Control Group

When analyzing only participants who did not receive any intervention in the MAPT study (decreasing sample to 122 participants, suggesting reduced power) and using the cutoff of 0.107, 50 participants (41.0%) were considered Aβ+. This group presented no differences in descriptive characteristics compared with participants in the Aβ– group. Results given by mixed models remained similar for MMSE score, with participants in the Aβ+ group declining and participants in the Aβ– group remaining stable over time. Both groups (Aβ+ and Aβ–) presented within-group significant decline in composite cognitive score and ADCS-ADL score over time, but there was no significant between-group difference. CDR sum of boxes only worsened among the Aβ+ group, with no significant adjusted between-group mean difference (eTable 4 in the Supplement).

### Sensitivity Analysis With Plasma Aβ_42/40_ as a Continuous Variable

Analyzed as a continuous variable, plasma Aβ_42/40_ was positively associated with the composite cognitive score during follow-up, indicating that participants with lower plasma Aβ_42/40_ had a more pronounced decline in composite cognitive score over time (adjusted β = 5.51; 95% CI, 1.35 to 9.67; *P* = .009), but results were not statistically significant after additionally adjusting for APOE ε4 genotype (β = 4.22; 95% CI, −0.17 to 8.62; *P* = .06). Significant associations were also observed for MMSE score in the adjusted model not including APOE ε4 genotype (adjusted β = 18.32; 95% CI, 3.44 to 33.20; *P* = .02). However, no significant associations were observed for all outcomes at the end of follow-up in the model additionally adjusted for APOE ε4 genotype (eTable 5 in the Supplement).

## Discussion

This study investigated the association between plasma Aβ_42/40_ status and cognitive decline among community-dwelling older adults and found that low plasma Aβ_42/40_ was associated with more pronounced decline in cognitive function during a median follow-up of 3.9 years. However, this biomarker was not associated with changes in ADCS-ADL score. Results were confirmed with an alternative cutoff. Sensitivity analysis restricted to the control group of MAPT Study confirmed the main findings for MMSE score.

These important findings are in line with previous investigations on the topic. The first longitudinal studies exploring plasma Aβ measures and outcomes of cognitive function among older adults associated low plasma Aβ_42/40_ with greater risk of MCI or AD after a median follow-up of 3.7 years^13^ and with more pronounced cognitive decline over 9 years.^16^ Accordingly, high plasma Aβ_40/42_ (the inverse ratio) was associated with more pronounced decline in global cognition during a 10-year period among older women volunteers from the Nurses’ Health Study.^14^ In contrast, high baseline plasma Aβ_42_ and Aβ_40_ values were associated with faster decline in multiple cognitive domains among a sample of older adults followed for approximately 4.5 years.^15^ However, not all studies were able to identify longitudinal associations between plasma Aβ and cognitive decline or conversion to MCI and AD.^29,30^ Comparisons of the current findings with older publications should nevertheless be cautious, given that the lack of sensitive and accurate analytical methods precluded high individual accuracy and achieving consistent and reliable evidence with the prior assay measurements.

More recently, the association between plasma Aβ and clinical cognitive outcomes has been explored with improved techniques for assessing plasma Aβ in some cross-sectional studies, which identified mixed findings among older adults.^31,32,33^ Highlighting the need for determining early predictors of cognitive impairment, it is imperative to explore such associations longitudinally, as we did in the present study. Despite the few reports focusing on cognitive outcomes and evaluating participants with different cognitive status and age ranges, the existing evidence from recent longitudinal studies points toward the usefulness of plasma Aβ_42/40_.^17,18^ The plasma Aβ_42/40_ ratio was not investigated by Iulita et al,^19^ but authors found that lower plasma Aβ_42_ and Aβ_40_ alone were both associated with a 3-year cognitive decline among a cohort of at-risk individuals and individuals clinically diagnosed with probable AD. On the other hand, a study of patients with AD found no association between plasma Aβ_42/40_ and MMSE score after a 2-year follow-up.^20^

Taken together, evidence from our study suggests that plasma Aβ_42/40_ is capable of identifying later cognitive decline among community-dwelling older adults with spontaneous memory concerns. Although this field is still in its beginning, our findings support the potential utility of plasma Aβ in research (eg, for selection of at-risk individuals for clinical trials or use as a proxy end point alongside other clinical markers). The usefulness of this biomarker in clinical care (ie, to increase diagnostic confidence, determine therapeutic strategies, or provide additional information on the brain Aβ deposition status of individuals) nevertheless demands further investigations.

### Strengths and Limitations

This study has multiple strengths. We assessed multiple cognitive outcomes and used a recent and improved measurement technique for plasma Aβ. Moreover, the longitudinal approach and the relatively large sample size are additional strengths. However, there are some limitations to be mentioned. This was a secondary analysis of a randomized clinical trial. However, the MAPT intervention did not affect cognition,^22^ and allocation to intervention groups was included as a confounder in the analyses. Plasma Aβ peptides were only assessed in a subsample of participants from MAPT, 1 or 2 years after inclusion because baseline samples were not available. Some characteristics of MAPT participants at inclusion were not similar between the sample of the present study and those who were not included, what may potentially be a selection bias. As normally seen in long follow-up studies, measures were not available to all participants at all moments. In addition, the sensitivity analysis restricted to the control group of MAPT was performed with a smaller sample and thus presented limited statistical power; its results should be therefore interpreted with caution. Finally, it is worth mentioning that participants of this study presented a particularly high educational level.

## Conclusions

With life expectancy increasing worldwide, interest in identifying early markers of cognitive decline has gained momentum, putting biomarkers with a potential to predict cognitive impairment in the spotlight. In the present study, low plasma Aβ_42/40_ was longitudinally associated with more pronounced declines in cognitive function, measured by multiple outcomes, during as long as 4 years of follow-up among community-dwelling older adults. Following evidence from previous publications^2,3,4,5^ that central and peripheral Aβ load are in dynamic balance, our findings show that plasma Aβ_42/40_ may be used to identify people at risk of cognitive decline, being an alternative to more complex and expensive measures such as PET scanning or CSF Aβ load. General cutoffs for determining plasma Aβ status remain to be determined in future investigations. Further studies with long follow-up periods and that target multiple cognitive measures are needed to confirm its utility in clinical practice and public health care.

## Notes

Supplement. **eTable 1. **Comparison of Characteristics at First Visit of MAPT Study (Original Baseline) for Participants Included and Not Included in the Present Study **eTable 2. **Mixed-Effect Linear Regression Analysis for Variation in Outcomes Over Time According to Plasma Amyloid-β42/40 Status Among Community-Dwelling Older Adults; Sensitivity Analysis Without Adjustment for APOE ε4 Genotype **eTable 3. **Mixed-Effect Linear Regression Analysis for Variation in Outcomes Over Time According to Plasma Amyloid-β42/40 Status Among Community-Dwelling Older Adults; Sensitivity Analysis Using 25th Percentile of Plasma Amyloid-β42/40 as the New Cutoff **eTable 4. **Mixed-Effect Linear Regression Analysis for Variation in Outcomes Over Time According to Plasma Amyloid-β42/40 Status Among Community-Dwelling Older Adults; Sensitivity Analysis Restricted to the Placebo Group of MAPT Study (n=122) **eTable 5. **Mixed-Effect Linear Regression Analysis for Associations Between Plasma Amyloid-β42/40 as a Continuous Variable and Outcomes Over Time Among Community-Dwelling Older Adults
