# Supplementary material for: Assessment of Plasma Amyloid-β42/40 and Cognitive Decline Among Community-Dwelling Older Adults
Source: JAMA Netw Open. 2020 Dec 17;3(12):e2028634. doi: 10.1001/jamanetworkopen.2020.28634 (PMC7747018; doi:10.1001/jamanetworkopen.2020.28634)
Supplement: Supplement. — eTable 1. Comparison of Characteristics at First Visit of MAPT Study (Original Baseline) for Participants Included and Not Included in the Present Study eTable 2. Mixed-Effect Linear Regression Analysis for Variation in Outcomes Over Time According to Plasma Amyloid-β42/40 Status Among Community-Dwelling Older Adults; Sensitivity Analysis Without Adjustment for APOE ε4 Genotype eTable 3. Mixed-Effect Linear Regression Analysis for Variation in Outcomes Over Time According to Plasma Amyloid-β42/40 Status Among Community-Dwelling Older Adults; Sensitivity Analysis Using 25th Percentile of Plasma Amyloid-β42/40 as the New Cutoff eTable 4. Mixed-Effect Linear Regression Analysis for Variation in Outcomes Over Time According to Plasma Amyloid-β42/40 Status Among Community-Dwelling Older Adults; Sensitivity Analysis Restricted to the Placebo Group of MAPT Study (n=122) eTable 5. Mixed-Effect Linear Regression Analysis for Associations Between Plasma Amyloid-β42/40 as a Continuous Variable and Outcomes Over Time Among Community-Dwelling Older Adults [file jamanetwopen-e2028634-s001.pdf]

Supplemental Online Content

Giudici KV, de Souto Barreto P, Guyonnet S, Li Y, Bateman RJ, Vellas B; MAPT/DSA Group. Assessment of plasma amyloid-$\beta_{42/40}$ and cognitive decline among community-dwelling older adults. *JAMA Netw Open*. 2020;3(12):e2028634. doi:10.1001/jamanetworkopen.2020.28634

eTable 1. Comparison of Characteristics at First Visit of MAPT Study (Original Baseline) for Participants Included and Not Included in the Present Study

eTable 2. Mixed-Effect Linear Regression Analysis for Variation in Outcomes Over Time According to Plasma Amyloid-β42/40 Status Among Community-Dwelling Older Adults; Sensitivity Analysis Without Adjustment for APOE ε4 Genotype

eTable 3. Mixed-Effect Linear Regression Analysis for Variation in Outcomes Over Time According to Plasma Amyloid-β42/40 Status Among Community-Dwelling Older Adults; Sensitivity Analysis Using 25th Percentile of Plasma Amyloid-β42/40 as the New Cutoff

eTable 4. Mixed-Effect Linear Regression Analysis for Variation in Outcomes Over Time According to Plasma Amyloid-β42/40 Status Among Community-Dwelling Older Adults; Sensitivity Analysis Restricted to the Placebo Group of MAPT Study (n=122)

eTable 5. Mixed-Effect Linear Regression Analysis for Associations Between Plasma Amyloid-β42/40 as a Continuous Variable and Outcomes Over Time Among Community-Dwelling Older Adults

This supplemental material has been provided by the authors to give readers additional information about their work.

© 2020 Giudici KV et al. *JAMA Network Open.*

eTable 1. Comparison of Characteristics at First Visit of MAPT Study (Original Baseline) for Participants Included and Not Included in the Present Study

	**Total**	**Included**	**Not included**
	**n = 1,679**	**n = 483**	**n = 1,196**
	**median (IQR)***	**median (IQR)***	**median (IQR)***
Female sex	1087 (64.7%)	286 (59.2%)	801 (67.0%)†
Age (years)	75.0 (72.0-78.0)	75.0 (72.0-79.0)	74.0 (72.0-78.0)†
Education (n = 1,643)			
No diploma	85 (5.2%)	22 (4.6%)	63 (5.4%)
Primary school certificate	286 (17.4%)	99 (20.8%)	187 (16.0%)
Secondary education	553 (33.7%)	158 (33.2%)	395 (33.9%)
High school diploma	242 (14.7%)	69 (14.5%)	173 (14.8%)
University level	477 (29.0%)	128 (26.9%)	349 (29.9%)
Weight (kg) (n = 1,672)	67.9 (59.0-77.0)	69.0 (60.0-79.0)	67.0 (58.0-76.0)†
Body mass index (kg/m^2) (n = 1,672)	25.8 (23.2-28.5)	25.9 (23.3-28.4)	25.8 (23.2-28.6)
**Clinical tests**			
Composite cognitive score**	0.08 (−0.38 to 0.45)	0.04 (−0.46 to 0.37)	0.09 (−0.35 to 0.47)†
CDR sum of boxes (range 0–18) (n = 1,678)	0.0 (0-0.5)	0.0 (0-0.5)	0.0 (0-0.5)†
CDR status (n=1,678)			
Cognitively normal (CDR score = 0)	972 (57.9%)	256 (53.0%)	716 (59.9%)†
Mild cognitive impairment (CDR score = 0.5)	706 (42.1%)	227 (47.0%)	479 (40.1%)
MMSE score (range 0–30)	28 (27-29)	28 (27-29)	28 (27-29)
ADCS-ADL score (range 0–45) (n = 1,665)	41 (37-43)	40 (37-43)	41 (38-44)†
Geriatric Depression Scale score (range 0–15) (n = 1,670)	3 (1-4)	2 (1-4)	3 (1-5)†
**ApoE ε4 genotype (n = 1,300)**			
ApoE ε4-carriers	299 (23.0%)	121 (27.6%)	178 (20.6%)†
Non-ApoE ε4-carriers	1001 (77.0%)	317 (72.4%)	684 (79.4%)
**Allocation to MAPT groups**			
Multidomain intervention plus ω-3	417 (24.8%)	129 (26.7%)	288 (24.1%)
ω-3 supplementation alone	422 (25.1%)	112 (23.2%)	310 (25.9%)
Multidomain intervention plus placebo	420 (25.0%)	120 (24.8%)	300 (25.1%)
Placebo alone	420 (25.0%)	122 (25.3%)	298 (24.9%)

© 2020 Giudici KV et al. *JAMA Network Open.*

Data presented in this table correspond for the original inclusion of MAPT study (not the baseline considered in main analysis of this manuscript); ADCS-ADL, Alzheimer's Disease Cooperative Study - Activities of Daily Living; ApoE, apolipoprotein E; CDR, Clinical Dementia Rating; IQR, interquartile range; MAPT, Multidomain Alzheimer Preventive Trial; MMSE, Mini-Mental State Examination; *Except where indicated other; **based on the Z-score of four cognitive tests (free and total recall of the Free and Cued Selective Reminding test, ten Mini-Mental State Examination orientation items, Digit Symbol Substitution Test and Category Naming Test); † $p<0.05$ based on Wilcoxon-Mann-Whitney test or Pearson's Chi-Square test.

© 2020 Giudici KV et al. *JAMA Network Open.*

eTable 2. Mixed-Effect Linear Regression Analysis for Variation in Outcomes Over Time According to Plasma Amyloid-β42/40 Status Among Community-Dwelling Older Adults; Sensitivity Analysis Without Adjustment for APOE ε4 Genotype

	**Estimated mean within-group change from the 12-month visit* (95% CI); P-value**				**Between-group difference between low and normal plasma amyloid-$\beta_{42/40}$ over time (95% CI); P-value**			
	**Low plasma amyloid-$\beta_{42/40}$****		**Normal plasma amyloid-$\beta_{42/40}$**		**Unadjusted model*****		**Adjusted model******	
Composite cognitive score****								
24 months (1-year change)	-0.25 (-0.33, -0.16);	**<0.001**	-0.12 (-0.19, -0.06);	**<0.001**	-0.12 (-0.23, -0.02);	**0.03**	-0.14 (-0.24, -0.03);	**0.01**
36 months (2-year change)	-0.35 (-0.44, -0.26);	**<0.001**	-0.16 (-0.22, -0.09);	**<0.001**	-0.19 (-0.30, -0.08);	**0.001**	-0.20 (-0.31, -0.09);	**<0.001**
48 months (3-year change)	-0.38 (-0.48, -0.28);	**<0.001**	-0.19 (-0.26, -0.12);	**<0.001**	-0.19 (-0.31, -0.07);	**0.002**	-0.21 (-0.34, -0.08);	**0.001**
60 months (4-year change)	-0.45 (-0.56, -0.35);	**<0.001**	-0.26 (-0.33, -0.19);	**<0.001**	-0.20 (-0.32, -0.07);	**0.002**	-0.24 (-0.37, -0.11);	**<0.001**
CDR sum of boxes (range 0–18)								
24 months (1-year change)	0.26 (0.10, 0.41);	**0.002**	0.08 (-0.04, 0.19);	0.18	0.18 (-0.02, 0.37);	0.07	0.12 (-0.05, 0.30);	0.17
36 months (2-year change)	0.31 (0.15, 0.48);	**<0.001**	0.12 (0.01, 0.24);	**0.04**	0.19 (-0.01, 0.39);	0.06	0.18 (-0.01, 0.36);	0.06
48 months (3-year change)	0.29 (0.10, 0.47);	**0.002**	0.10 (-0.02, 0.22);	0.11	0.19 (-0.04, 0.41);	0.10	0.15 (-0.05, 0.36);	0.15
60 months (4-year change)	0.43 (0.24, 0.62);	**<0.001**	0.29 (0.16, 0.41);	**<0.001**	0.15 (-0.08, 0.37);	0.21	0.20 (0.01, 0.41);	0.06
MMSE score (range 0–30)								
24 months (1-year change)	-0.47 (-0.78, -0.16);	**0.003**	0.03 (-0.20, 0.25);	0.82	-0.50 (-0.88, -0.11);	**0.01**	-0.56 (-0.95, -0.18);	**0.004**
36 months (2-year change)	-0.69 (-1.01, -0.36);	**<0.001**	-0.11 (-0.34, 0.12);	0.34	-0.57 (-0.97, -0.18);	**0.004**	-0.66 (-1.06, -0.27);	**0.001**
48 months (3-year change)	-0.37 (-0.74, -0.01);	**0.005**	-0.01 (-0.25, 0.23);	0.94	-0.36 (-0.80, 0.07);	0.10	-0.38 (-0.83, 0.08);	0.10

© 2020 Giudici KV et al. *JAMA Network Open.*

60 months (4-year change)	-0.72 (-1.10, -0.35);	**<0.001**	-0.16 (-0.41, 0.08);	0.20	-0.56 (-1.01, -0.11);	**0.01**	-0.74 (-1.20, -0.28);	**0.002**
ADCS-ADL score (range 0–45)								
24 months (1-year change)	-1.39 (-2.16, -0.62);	**<0.001**	-0.50 (-1.05, 0.06);	0.08	-0.89 (-1.84, 0.06);	0.06	-0.48 (-1.42, 0.46);	0.32
36 months (2-year change)	-1.28 (-2.09, -0.48);	**0.002**	-0.22 (-0.78, 0.35);	0.46	-1.07 (-2.05, -0.08);	**0.03**	-0.91 (-1.88, 0.07);	0.07
48 months (3-year change)	-1.99 (-2.90, -1.07);	**<0.001**	-0.61 (-1.21, -0.01);	**0.05**	-1.37 (-2.47, -0.28);	**0.01**	-0.96 (-2.08, 0.16);	0.09
60 months (4-year change)	-1.73 (-2.67, -0.78);	**<0.001**	-0.85 (-1.46, -0.24);	**0.006**	-0.88 (-2.00, 0.25);	0.13	-0.20 (-1.34, 0.94);	0.73

ADCS-ADL, Alzheimer's Disease Cooperative Study - Activities of Daily Living; CDR, Clinical Dementia Rating; CI, confidence interval; MMSE, Mini-Mental State Examination; *Outcomes evolution was compared considering the moment when plasma amyloid-β was measured as the baseline (12 months for 92.8% of the sample and 24 months for 7.2%). Negative values for within-group differences mean cognitive decline, except for CDR sum of boxes (for which it is given by positive values). Positive values for between-group differences indicate more pronounced cognitive decline among the low plasma $A\beta_{42/40}$ group, except for CDR sum of boxes (for which it is given by negative values); **Low $A\beta_{42/40}$ defined as ≤ 0.107; ***N=481; ****N=476, model adjusted by age, sex, education, body mass index, Geriatric Depression Scale score, MAPT intervention groups and CDR status at baseline (this last, except for the analysis with CDR sum of boxes); ****Based on the Z-score of four cognitive tests (free and total recall of the Free and Cued Selective Reminding test, ten Mini-Mental State Examination orientation items, Digit Symbol Substitution Test and Category Naming Test).

© 2020 Giudici KV et al. *JAMA Network Open.*

eTable 3. Mixed-Effect Linear Regression Analysis for Variation in Outcomes Over Time According to Plasma Amyloid-β42/40 Status Among Community-Dwelling Older Adults; Sensitivity Analysis Using 25th Percentile of Plasma Amyloid-β42/40 as the New Cutoff

	**Estimated mean within-group change from the 12-month visit* (95% CI); P-value**				**Between-group difference between low and normal plasma amyloid-$\beta_{42/40}$ over time* (95% CI); P-value**			
	**Low plasma amyloid-$\beta_{42/40}$******		**Normal plasma amyloid-$\beta_{42/40}$**		**Unadjusted model*****		**Adjusted model******	
Composite cognitive score****								
24 months (1-year change)	-0.25 (-0.35, -0.15);	**<0.001**	-0.14 (-0.19, -0.08);	**<0.001**	-0.12 (-0.24, 0.00);	**0.04**	-0.07 (-0.19, 0.05);	0.27
36 months (2-year change)	-0.29 (-0.40, -0.19);	**<0.001**	-0.20 (-0.26, -0.14);	**<0.001**	-0.10 (-0.22, 0.03);	0.12	-0.06 (-0.19, 0.06);	0.33
48 months (3-year change)	-0.34 (-0.46, -0.22);	**<0.001**	-0.22 (-0.29, -0.16);	**<0.001**	-0.12 (-0.25, 0.02);	0.09	-0.10 (-0.24, 0.04);	0.16
60 months (4-year change)	-0.46 (-0.58, -0.33);	**<0.001**	-0.28 (-0.34, -0.21);	**<0.001**	-0.18 (-0.32, -0.04);	**0.01**	-0.18 (-0.33, -0.03);	**0.02**
CDR sum of boxes (range 0–18)								
24 months (1-year change)	0.28 (0.10, 0.46);	**0.003**	0.09 (-0.02, 0.19);	0.11	0.19 (-0.02, 0.40);	0.07	0.12 (-0.08, 0.32);	0.25
36 months (2-year change)	0.32 (0.13, 0.51);	**0.001**	0.14 (0.03, 0.25);	**0.01**	0.18 (-0.04, 0.40);	0.11	0.14 (-0.07, 0.35);	0.20
48 months (3-year change)	0.31 (0.10, 0.53);	**0.004**	0.11 (0.00, 0.23);	0.06	0.20 (-0.04, 0.44);	0.11	0.14 (-0.10, 0.38);	0.25
60 months (4-year change)	0.61 (0.39, 0.83);	**<0.001**	0.26 (0.14, 0.37);	**<0.001**	0.35 (0.10, 0.60);	**0.006**	0.37 (0.13, 0.62);	**0.003**
MMSE score (range 0–30)								
24 months (1-year change)	-0.48 (-0.84, -0.13);	**0.008**	-0.02 (-0.23, 0.19);	0.83	-0.46 (-0.88, -0.05);	**0.03**	-0.26 (-0.68, 0.16);	0.23
36 months (2-year change)	-0.57 (-0.95, -0.20);	**0.003**	-0.21 (-0.42, 0.01);	0.06	-0.36 (-0.80, 0.07);	0.10	-0.25 (-0.69, 0.19);	0.27

© 2020 Giudici KV et al. *JAMA Network Open.*

48 months (3-year change)	-0.29 (-0.72, 0.13);	0.18	-0.07 (-0.30, 0.16);	0.54	-0.22 (-0.70, 0.26);	0.37	-0.09 (-0.60, 0.41);	0.71
60 months (4-year change)	-0.74 (-1.18, -0.30);	**0.001**	-0.22 (-0.45, 0.01);	0.07	-0.52 (-1.02, -0.02);	**0.04**	-0.53 (-1.04, -0.01);	**0.05**
ADCS-ADL score (range 0–45)								
24 months (1-year change)	-1.68 (-2.56, -0.79);	**<0.001**	-0.50 (-1.02, 0.02);	0.06	-1.18 (-2.21, -0.16);	**0.02**	-0.67 (-1.71, 0.38);	0.21
36 months (2-year change)	-1.21 (-2.14, -0.28);	**0.01**	-0.35 (-0.89, 0.18);	0.20	-0.86 (-1.93, 0.21);	0.12	-0.47 (-1.56, 0.63);	0.40
48 months (3-year change)	-2.26 (-3.32, -1.21);	**<0.001**	-0.67 (-1.24, -0.10);	**0.02**	-1.60 (-2.79, -0.40);	**0.009**	-0.94 (-2.19, 0.31);	0.14
60 months (4-year change)	-1.96 (-3.07, -0.86);	**0.001**	-0.88 (-1.46, -0.30);	**0.003**	-1.09 (-2.33, 0.16);	0.09	-0.43 (-1.72, 0.86);	0.51

ADCS-ADL, Alzheimer's Disease Cooperative Study - Activities of Daily Living; CDR, Clinical Dementia Rating; CI, confidence interval; MMSE, Mini-Mental State Examination; *Evolution of outcomes was compared considering the moment when plasma amyloid-β was measured as the baseline (12 months for 92.8% of the sample and 24 months for 7.2%). Negative values for within-group differences mean cognitive decline, except for CDR sum of boxes (for which it is given by positive values). Positive values for between-group differences indicate more pronounced cognitive decline among the low plasma $A\beta_{42/40}$ group, except for CDR sum of boxes (for which it is given by negative values); ** Low $A\beta_{42/40}$ defined as ≤0.1034877 (lowest quartile); ***N=481; ****N=433, model adjusted by age, sex, education, body mass index, Geriatric Depression Scale score, ApoE ε4 genotype, MAPT intervention groups and CDR status at baseline (this last, except for the analysis with CDR sum of boxes); ****Based on the Z-score of four cognitive tests (free and total recall of the Free and Cued Selective Reminding test, ten Mini-Mental State Examination orientation items, Digit Symbol Substitution Test and Category Naming Test).

© 2020 Giudici KV et al. *JAMA Network Open.*

eTable 4. Mixed-Effect Linear Regression Analysis for Variation in Outcomes Over Time According to Plasma Amyloid-β42/40 Status Among Community-Dwelling Older Adults; Sensitivity Analysis Restricted to the Placebo Group of MAPT Study (n=122)

	**Estimated mean within-group change from the 12-month visit* (95% CI); P-value**				**Between-group difference between low and normal plasma amyloid-$\beta_{42/40}$ over time* (95% CI); P-value**			
	**Low plasma amyloid-$\beta_{42/40}$****		**Normal plasma amyloid-$\beta_{42/40}$**		**Unadjusted model*****		**Adjusted model******	
Composite cognitive score****								
24 months (1-year change)	-0.21 (-0.38, -0.03);	**0.01**	-0.12 (-0.27, 0.03);	0.11	-0.09 (-0.31, 0.14);	0.45	-0.01 (-0.23, 0.21);	0.93
36 months (2-year change)	-0.41 (-0.59, -0.22);	**<0.001**	-0.20 (-0.34, -0.05);	**0.009**	-0.21 (-0.44, 0.02);	0.08	-0.18 (-0.41, 0.04);	0.11
48 months (3-year change)	-0.36 (-0.56, -0.16);	**<0.001**	-0.17 (-0.33, -0.01);	**0.04**	-0.19 (-0.45, 0.06);	0.14	-0.06 (-0.31, 0.20);	0.65
60 months (4-year change)	-0.37 (-0.58, -0.16);	**0.001**	-0.26 (-0.43, -0.10);	**0.001**	-0.11 (-0.37, 0.16);	0.42	-0.05 (-0.32, 0.22);	0.72
CDR sum of boxes (range 0–18)								
24 months (1-year change)	0.24 (-0.13, 0.60);	0.20	0.02 (-0.28, 0.33);	0.88	0.21 (-0.26, 0.69);	0.38	0.13 (-0.29, 0.56);	0.54
36 months (2-year change)	0.47 (0.10, 0.85);	**0.01**	0.01 (-0.30, 0.31);	0.96	0.47 (-0.02, 0.95);	0.06	0.45 (0.01, 0.89);	**0.04**
48 months (3-year change)	0.31 (-0.10, 0.72);	0.14	0.11 (-0.22, 0.43);	0.53	0.20 (-0.32, 0.73);	0.45	0.10 (-0.39, 0.58);	0.70
60 months (4-year change)	0.57 (0.14, 0.99);	**0.009**	0.31 (-0.02, 0.64);	0.07	0.26 (-0.28, 0.79);	0.35	0.42 (-0.08, 0.91);	0.10
MMSE score (range 0–30)								
24 months (1-year change)	-0.66 (-1.20, -0.11);	**0.02**	0.31 (-0.16, 0.77);	0.20	-0.96 (-1.68, -0.25);	**0.009**	-1.01 (-1.73, -0.28);	**0.007**
36 months (2-year change)	-1.05 (-1.61, -0.48);	**<0.001**	-0.14 (-0.61, 0.32);	0.55	-0.90 (-1.64, -0.17);	**0.02**	-1.19 (-1.94, -0.45);	**0.002**

© 2020 Giudici KV et al. *JAMA Network Open.*

48 months (3-year change)	-0.26 (-0.89, 0.37);	0.41	0.25 (-0.25, 0.74);	0.33	-0.51 (-1.31, -0.29);	0.21	-0.41 (-1.25, 0.43);	0.33
60 months (4-year change)	-0.87 (-1.51, -0.22);	**0.008**	0.04 (-0.47, 0.55);	0.89	-0.91 (-1.73, -0.08);	**0.03**	-1.22 (-2.08, -0.36);	**0.006**
ADCS-ADL score (range 0–45)								
24 months (1-year change)	-1.64 (-3.11, -0.18);	**0.03**	-0.76 (-2.05, 0.54);	0.25	-0.89 (-2.84, 1.07);	0.37	-0.22 (-2.19, 1.74);	0.82
36 months (2-year change)	-1.59 (-3.11, -0.06);	**0.04**	-0.26 (-1.55, 1.03);	0.70	-1.33 (-3.33, 0.67);	0.19	-0.69 (-2.71, 1.32);	0.50
48 months (3-year change)	-2.35 (-4.06, -0.63);	**0.007**	-1.73 (-3.12, -0.35);	**0.01**	-0.61 (-2.81, 1.59);	0.59	0.07 (-2.21, 2.35);	0.95
60 months (4-year change)	-1.94 (-3.67, -0.21);	**0.03**	-1.63 (-3.03, -0.23);	**0.02**	-0.31 (-2.54, 1.92);	0.79	0.29 (-2.01, 2.58);	0.81

ADCS-ADL, Alzheimer's Disease Cooperative Study - Activities of Daily Living; CDR, Clinical Dementia Rating; CI, confidence interval; MMSE, Mini-Mental State Examination; *Outcomes evolution was compared considering the moment when plasma amyloid-β was measured as the baseline (12 months for 92.8% of the sample and 24 months for 7.2%). Negative values for within-group differences mean cognitive decline, except for CDR sum of boxes (for which it is given by positive values). Positive values for between-group differences indicate more pronounced cognitive decline among the low plasma amyloid-$\beta_{42/40}$ group, except for CDR sum of boxes (for which it is given by negative values); **Low amyloid-$\beta_{42/40}$ defined as ≤ 0.107; ***N=122; ****N=111, model adjusted by age, sex, education, body mass index, Geriatric Depression Scale score, Apolipoprotein E ε4 genotype and CDR status at baseline (this last, except for the analysis with CDR sum of boxes); ****Based on four cognitive tests (free and total recall of the Free and Cued Selective Reminding test, ten Mini-Mental State Examination orientation items, Digit Symbol Substitution Test and Category Naming Test).

© 2020 Giudici KV et al. *JAMA Network Open.*

eTable 5. Mixed-Effect Linear Regression Analysis for Associations Between Plasma Amyloid-β42/40 as a Continuous Variable and Outcomes Over Time Among Community-Dwelling Older Adults

	**Unadjusted model***				**Adjusted model 1****			**Adjusted model 2*****		
	**β coefficient**** (95% CI)**		**p-value**		**β coefficient**** (95% CI)**		**p-value**	**β coefficient**** (95% CI)**		**p-value**
Composite cognitive score*****										
24 months (1-year change)	3.10	(-0.40, 6.59)	0.08		1.92	(-1.83, 5.68)	0.31	3.23	(-0.32, 6.78)	0.07
36 months (2-year change)	4.90	(1.26, 8.54)	**0.008**		3.79	(-0.15, 7.72)	0.06	5.29	(1.60, 8.97)	**0.005**
48 months (3-year change)	5.55	(1.65, 9.45)	**0.005**		4.21	(-0.19, 8.60)	0.06	5.52	(1.38, 9.67)	**0.009**
60 months (4-year change)	4.84	(0.81, 8.86)	**0.02**		4.22	(-0.17, 8.62)	0.06	5.51	(1.35, 9.67)	**0.009**
CDR sum of boxes										
24 months (1-year change)	-4.73	(-11.05, 1.60)	0.14		-2.59	(-8.92, 3.74)	0.42	-2.88	(-8.71, 2.95)	0.33
36 months (2-year change)	-2.77	(-9.30, 3.77)	0.41		-1.02	(-7.63, 5.59)	0.76	-2.06	(-8.09, 3.98)	0.50
48 months (3-year change)	-4.83	(-11.86, 2.20)	0.18		-0.97	(-8.32, 6.39)	0.80	-2.64	(-9.40, 4.12)	0.44
60 months (4-year change)	-4.39	(-11.62, 2.85)	0.23		-3.29	(-10.68, 4.10)	0.38	-4.22	(-11.03, 2.58)	0.22
MMSE score										
24 months (1-year change)	11.88	(-0.61, 24.38)	0.06		7.88	(-5.48, 21.23)	0.25	14.44	(1.75, 27.13)	**0.03**
36 months (2-year change)	16.93	(3.97, 29.89)	**0.01**		13.66	(-0.32, 27.63)	0.06	19.40	(6.22, 32.58)	**0.004**
48 months (3-year change)	12.12	(-1.81, 26.05)	0.09		8.16	(-7.45, 23.77)	0.31	12.78	(-2.01, 27.57)	0.09
60 months (4-year change)	13.36	(-1.00, 27.71)	0.07		12.49	(-3.16, 28.15)	0.12	18.32	(3.44, 33.20)	**0.02**
ADCS-ADL score										
24 months (1-year change)	27.91	(-3.20, 59.02)	0.08		12.26	(-20.80, 45.32)	0.47	10.96	(-20.25, 42.16)	0.49
36 months (2-year change)	36.07	(3.94, 68.20)	**0.03**		23.64	(-10.81, 58.09)	0.18	24.28	(-7.96, 56.53)	0.14
48 months (3-year change)	74.72	(40.17, 109.28)	**<0.001**		46.78	(8.26, 85.30)	**0.02**	51.61	(15.27, 87.94)	**0.005**
60 months (4-year change)	24.42	(-11.55, 60.39)	0.18		10.67	(-28.15, 49.49)	0.59	7.51	(-29.20, 44.22)	0.69

© 2020 Giudici KV et al. *JAMA Network Open.*

ADCS-ADL, Alzheimer's Disease Cooperative Study - Activities of Daily Living; CDR, Clinical Dementia Rating; CI, confidence interval; MMSE, Mini-Mental State Examination; *N=480, considering the moment when plasma amyloid-β was measured as the baseline (12 months or 24 months). **N=432, model adjusted by age, sex, education, body mass index, Geriatric Depression Scale score, Apolipoprotein E ε4 genotype, MAPT groups and CDR status at baseline (this last, except for the analysis with CDR sum of boxes); ***N=475, model adjusted by age, sex, education, body mass index, Geriatric Depression Scale score, MAPT groups and CDR status at baseline (this last, except for the analysis with CDR sum of boxes); ****For interaction between continuous plasma $A\beta_{42/40}$ and time. Positive values of β coefficient indicate more pronounced cognitive decline among the low plasma $A\beta_{42/40}$ group, except for CDR sum of boxes (for which it is given by negative values); *****Based on four cognitive tests (free and total recall of the Free and Cued Selective Reminding test, ten Mini-Mental State Examination orientation items, Digit Symbol Substitution Test and Category Naming Test).

© 2020 Giudici KV et al. *JAMA Network Open.*
